# Burnout syndrome in Brazil (2014–2024): regional variations and temporal trends in an epidemiological study

**DOI:** 10.47626/1679-4435-2025-1479

**Published:** 2025-09-14

**Authors:** Maria Fernanda Quandt Treml, Amanda Vieira Sarubbi, Maria Eduarda Smaniotto Madeira, Amanda Wollmann Rasoto, Nathalia Schwarzer

**Affiliations:** 1 Medicina, Universidade Regional de Blumenau, Blumenau, SC, Brasil.

**Keywords:** burnout psychological, Brazil, mental health, data interpretation, statistical, public health, esgotamento psicológico, Brasil, saúde mental, interpretação estatística de dados, saúde pública

## Abstract

**Introduction:**

Burnout syndrome is classified in International Classification of Diseases-11 as a disorder related to chronic workplace stress, characterized by emotional exhaustion, negative feelings toward work, and reduced professional efficacy. It represents a growing public health issue impacting quality of life and productivity.

**Objectives:**

To analyze the regional and temporal distribution of burnout syndrome in Brazil between 2014 and 2024, based on sociodemographic and clinical data available in the public health system.

**Methods:**

A retrospective, observational, quantitative study using data extracted from the Departamento de Informática do Sistema Único de Saúde system. Variables analyzed included sex, age group, race, substance use, psychotropic drug use, treatment regimen, referral to Psychosocial Care Centers, and clinical evolution. Statistical analysis was performed using Microsoft Excel 2013 with analysis of variance, 95%CI, and p < 0.05 as significance threshold.

**Results:**

Most reported cases were women (71.6%), aged 35 to 49 years (56.7%). The Southeast (52.8%) and Northeast (29.8%) regions concentrated most notifications. The year 2024 had the highest notification rate (28.4%; p = 0.008). Psychotropic drug use was identified in 51.7% of cases, while alcohol, tobacco, and illicit drug use accounted for less than 8%, without statistical significance. Conclusions: The syndrome showed a 96.4% increase over the analyzed period, predominantly affecting women in the Southeast region. This rise may be related to increased diagnostic awareness, professional training, and mental health awareness strategies.

## Abstract (pt)

**Introdução:**

A síndrome de *burnout* é classificada pela Classificação Internacional de Doenças-11 como um distúrbio relacionado ao estresse crônico no ambiente de trabalho, caracterizando-se por exaustão emocional, sentimentos de negativismo em relação ao trabalho e redução da eficácia profissional. Representa um problema crescente de saúde pública, com impactos na qualidade de vida e produtividade.

**Objetivos:**

Analisar a distribuição regional e temporal da síndrome de *burnout* no Brasil entre 2014 e 2024, com base em dados sociodemográficos e clínicos disponíveis em sistema público de saúde.

**Métodos:**

Estudo observacional, retrospectivo e quantitativo, com dados extraídos do sistema Departamento de Informática do Sistema Único de Saúde. Foram analisadas variáveis como sexo, faixa etária, raça, uso de substâncias, psicofármacos, regime de tratamento, encaminhamento para Centros de Atenção Psicossocial e evolução clínica. A análise estatística foi realizada no Microsoft Excel 2013, utilizando *analysis of variance* com intervalo de confiança de 95% e p < 0,05 como critério de significância.

**Resultados:**

A maioria dos casos notificados ocorreu em mulheres (71,6%) na faixa etária de 35 a 49 anos (56,7%). As regiões Sudeste (52,8%) e Nordeste (29,8%) concentraram os maiores registros. O ano de 2024 apresentou o pico de notificações (28,4%; p = 0,008). O uso de psicofármacos foi identificado em 51,7% dos casos, enquanto álcool, tabaco e drogas ilícitas representaram percentuais menores (< 8%), sem significância estatística.

**Conclusões:**

A síndrome apresentou crescimento expressivo (96,4%) no período analisado, com predomínio em mulheres da Região Sudeste. O aumento pode estar relacionado a maior sensibilidade diagnóstica, capacitação profissional e estratégias de conscientização em saúde mental.

## Introduction

Burnout syndrome was first described by psychologist Herbert Freudenberger in 1974, when he observed patterns of emotional exhaustion, depersonalization, and reduced professional accomplishment among individuals exposed to adverse work environments. In the 1980s, Christina Maslach and Susan Jackson expanded on the concept by developing the Maslach Burnout Inventory (MBI), the main tool used to measure burnout in international research. Their work consolidated diagnostic criteria and facilitated comparisons across studies.^^1^,^2^^

Due to its growing prevalence, burnout syndrome was included in the International Classification of Diseases, 11th revision (ICD-11) in 2019, categorized as “problems associated with employment or unemployment” (code QD85). The syndrome is recognized as an occupational phenomenon resulting from chronic workplace stress that has not been successfully managed, marking a significant shift in the global understanding of the condition.^^3^,^4^^

In Brazil, the situation is alarming: the country ranks second worldwide in the prevalence of occupational burnout, according to data from the International Stress Management Association (ISMA). This context is aggravated by factors such as work overload, excessive competitiveness, lack of institutional psychological support, economic instability, and the effects of the COVID-19 pandemic.^^5^-^8^^

Globally, occupational stress is associated with the loss of approximately 12 billion workdays per year, generating an estimated cost of US$ 1 trillion for the world economy due to decreased productivity, according to the World Health Organization (WHO) and the International Labour Organization (ILO).^^9^^

The main consequences of burnout syndrome include increased use of psychoactive substances — such as alcohol, tobacco, and psychotropic drugs — and a marked reduction in workplace productivity. These repercussions contribute to a rise in sick leaves due to mental and behavioral disorders, placing a growing financial burden on the Brazilian social security system through disability-related benefits.^^10^-^13^^

Despite the relevance of this issue, Brazilian research reveals significant gaps, especially in epidemiological analyses at the regional level based on official data. Most studies have focused on qualitative approaches or on specific occupational groups, such as physicians and teachers, limiting the understanding of the phenomenon at the national level.^^14^^

The present study aims to analyze the regional distribution of burnout syndrome in Brazil between 2014 and 2024 through an ecological and quantitative study using secondary data from the Departamento de Informática do Sistema Único de Saúde (DATASUS). It seeks to describe regional patterns of the syndrome and discuss their relationship with social and cultural determinants. The expected results are intended to support the strengthening of public policies for prevention, early diagnosis, and comprehensive mental health care, particularly in primary care. This approach aims to contribute to the planning of effective strategies to address the syndrome, aligned with the guidelines of the Brazilian Unified Health System (SUS) and national mental health policies.

## Methods

### Methodological strategy and data source

This was a quantitative study with an ecological and descriptive design, based on publicly available secondary data. Data were collected in February 2025 from the DATASUS platform. Records coded as ICD-10 Z73.0, referring to burnout syndrome, were selected.

The study period covered the years 2014 to 2024, stratified by the major regions of the country (North, Northeast, Southeast, South, and Center-West). This time frame was chosen due to the availability of the most recent data and the possibility of observing trends over a decade. The ecological approach was adopted as it allows the analysis of aggregated data by region, facilitating the identification of collective patterns and territorial inequalities in the phenomenon under study.

### Context and sample delimitation

All notifications related to burnout syndrome recorded during the selected period were included. The study sample corresponded to all hospital admissions reported with a burnout diagnosis in the DATASUS system, stratified by geographic region and by the available sociodemographic and clinical variables. Variables analyzed included: year of notification, region of residence, age group, sex, race/skin color, alcohol use, use of psychoactive drugs, use of psychotropic drugs, tobacco use, treatment regimen, clinical outcome, and referral to a Psychosocial Care Center (Centro de Atenção Psicossocial, CAPS).

Cases with missing values for the variables of interest were excluded only from the specific analysis in which the variable was absent, in order to preserve the internal consistency of data.

### Data collection and organization procedures

Data collection was conducted through the manual selection of filters on the DATASUS platform, with extraction into Microsoft Excel spreadsheets. Filters applied included: years 2014 to 2024, region of residence, and complementary variables. Data were organized into tables for subsequent statistical analysis, ensuring traceability and reproducibility of the procedures adopted.

### Statistical analysis

Statistical analysis was performed using the data analysis tool in Microsoft Excel. A one-way analysis of variance (ANOVA) was applied to compare notification frequencies among Brazilian regions. Subsequently, Tukey’s multiple comparison test was used with a 95% CI to identify which groups showed significant differences from one another. A p-value < 0.05 was considered statistically significant. Results were presented in charts and tables, highlighting the statistical differences identified between regions over the study period.

### Ethical considerations

Since this study used exclusively publicly available secondary data with no possibility of identifying individual information, prior approval by a research ethics committee was not required, in accordance with Resolution No. 466/2012 of the Brazilian National Health Council.

## Results

A total of 1,458 cases of burnout syndrome were reported in Brazil between 2014 and 2024. Most notifications occurred in the Southeast region (52.81%), followed by the Northeast (29.77%), South (12.35%), and Center-West (2.88%), with the lowest number recorded in the North (2.19%). A progressive increase in notifications was observed, particularly from 2020 onward, reaching its peak in 2024 (28.40%; p = 0.008) (Table 1).

**Table 1:** Percentage distribution of reported cases of burnout syndrome by region, sex, race, and age group in Brazil (2014-2024)

Year/Region (%)	North	Northeast	Southeast	South	Center-West	Total
2014	0.00	0.41	0.21	0.27	0.14	1.03
2015	0.07	0.34	0.62	0.41	0.21	1.65
2016	0.14	0.48	0.69	0.27	0.00	1.58
2017	0.00	1.37	1.30	0.34	0.14	3.16
2018	0.07	1.51	1.30	0.21	0.07	3.16
2019	0.07	1.78	1.65	0.55	0.07	4.12
2020	0.07	0.82	1.58	0.41	0.14	3.02
2021	0.07	2.40	6.79	0.82	0.27	10.36
2022	0.34	3.57	9.53	1.92	0.21	15.57
2023	0.48	7.48	16.32	2.95	0.75	27.98
2024	0.89	9.60	12.83	4.18	0.89	28.40
Total	2.19	29.77	52.81	12.35	2.88	100
p-value						0.008
Region/Sex (%)	Male	Female				
North	0.27	1.92				
Northeast	9.47	20.30				
Southeast	14.81	38.00				
South	3.22	9.12				
Center-West	0.62	2.26				
Total	28.40	71.60				
p-value						0.27
Region/Race (%)	Missing	White	Black	Yellow	Brown	Indigenous
North	0.21	0.75	0.14	0.07	1.03	0.00
Northeast	8.30	8.57	2.06	0.27	10.49	0.07
Southeast	1.58	33.47	5.56	0.41	11.73	0.07
South	0.82	9.60	0.55	0.07	1.30	0.00
Center-West	0.41	1.10	0.14	0.00	1.23	0.00
Total	11.32	53.50	8.44	0.82	25.79	0.14
p-value						0.08
Region/Age (%) (years)	15-19	20-34	35-49	50-64	65-79	
North	0.00	0.55	1.17	0.48	0.00	
Northeast	0.14	8.30	17.42	3.91	0.00	
Southeast	0.27	12.89	31.34	8.23	0.07	
South	0.27	4.25	5.01	2.81	0.00	
Center-West	0.00	0.48	1.78	0.62	0.00	
Total	0.69	26.47	56.72	16.05	0.07	
p-value						0.06

Sex distribution revealed a predominance of females, who accounted for 71.60% of notifications, while males represented 28.40% of cases (p = 0.27). The highest proportions were observed in the Southeast (52.81%) and Northeast (29.77%) regions (Table 1).

Regarding race/skin color, most reported patients were White (53.50%), followed by Brown (25.79%), Black (8.44%), Yellow (0.82%), and Indigenous (0.14%). The variable was missing in 11.32% of the records. The distribution remained relatively stable across regions (p = 0.08) (Table 1).

The age group most affected by burnout was 35-49 years (56.72%), while the least affected was 65-79 years (0.07%), with only 1 case reported in the Southeast region (p = 0.06) (Table 1).

Only 7.13% of patients reported alcohol consumption, while 72.84% denied use and 20.03% had missing data for this variable. The highest proportion of users was observed in the Northeast region (3.29%) (p = 0.12) (Table 2).

**Table 2:** Regional distribution of alcohol, tobacco, psychotropic drug, and psychoactive drug use among reported cases of burnout syndrome in Brazil (2014-2024)

Region/Alcohol (%)	Missing	Yes	No
North	0.27	0.00	1.92
Northeast	8.71	3.29	17.76
Southeast	8.37	2.61	41.84
South	2.19	0.89	9.26
Center-West	0.48	0.34	2.06
Total	20.03	7.13	72.84
p-value			0.12
Region/Tobacco use (%)			
North	0.48	0.00	1.71
Northeast	9.81	0.82	19.14
Southeast	9.81	3.09	39.92
South	2.13	1.65	8.57
Center-West	0.62	0.21	2.06
Total	22.84	5.76	71.40
p-value			0.12
Region/Psychotropic drugs (%)			
North	0.21	1.03	0.96
Northeast	7.96	14.06	7.75
Southeast	6.38	32.30	14.13
South	2.06	3.22	7.06
Center-West	0.48	1.10	1.30
Total	17.08	51.71	31.21
p-value			0.42
Region/Psychoactive drugs (%)			
North	0.27	0.07	1.85
Northeast	9.60	1.58	18.59
Southeast	8.23	2.61	41.98
South	2.40	0.21	9.74
Center-West	0.41	0.21	2.26
Total	20.92	4.66	74.42
p-value			0.10

Regarding tobacco use, 5.76% reported use, 71.40% denied use, and 22.84% had missing information. The Southeast concentrated the highest prevalence of users (3.09%) (p = 0.12) (Table 2).

Psychotropic drug use was reported by 51.71% of patients, being more prevalent in the Southeast (32.30%) and Northeast (14.06%). On the other hand, 31.21% denied use, and 17.80% of notifications did not include this information (p = 0.42) (Table 2).

Psychoactive drug use was recorded in only 4.66% of cases, with the highest concentration in the Southeast (2.61%). Most patients denied use (74.42%), while 20.92% of data were missing (p = 0.10) (Table 2).

Regarding treatment regimen, outpatient care predominated (92.11%), followed by missing records (5.08%) and hospitalizations (2.81%) (p = 0.05) (Table 3).

**Table 3:** Treatment modality, care in Psychosocial Care Centers (CAPS), and clinical outcomes of reported cases of burnout syndrome in Brazil (2014-2024)

Region/Treatment regimen (%)	Missing		Inpatient		Outpatient		
North	0.48		0.21		1.51		
Northeast	1.78		0.69		27.30		
Southeast	1.78		1.58		49.45		
South	0.69		0.21		11.45		
Center-West	0.34		0.14		2.40		
Total	5.08		2.81		92.11		
p-value				0.05
Region/CAPS (%)	Missing		Yes		No		
North	0.14		1.78		0.27		
Northeast	6.38		15.78		7.61		
Southeast	5.62		26.20		20.99		
South	1.37		4.60		6.38		
Center-West	0.55		1.51		0.82		
Total	14.06		49.86		36.08		
p-value				0.39
Region/Clinical outcome (%)	Missing	Recovery	Recovery not confirmed	Temporary disability	Partial permanent disability	Total permanent disability	Other
North	0.41	0.07	0.34	1.03	0.14	0.00	0.21
Northeast	2.26	0.75	1.99	22.22	0.48	0.14	1.92
Southeast	5.76	0.96	3.02	39.30	0.96	0.14	2.67
South	2.47	0.41	2.88	5.14	0.21	0.00	1.23
Center-West	0.21	0.21	0.27	1.65	0.14	0.00	0.41
Total	11.11	2.40	8.50	69.34	1.92	0.27	6.45
p-value							0.02

In 49.86% of notifications, patients were referred for treatment at CAPS, while 36.08% were not referred and 14.06% of cases had missing information (p = 0.39) (Table 3).

The most prevalent clinical outcome was temporary disability (69.34%). Cases classified as recovery accounted for 2.40%, while 8.50% were considered unconfirmed recovery. Other outcomes included partial permanent disability (1.92%), total permanent disability (0.27%), and unspecified evolution (6.35%). There were 11.11% missing records (p = 0.02) (Table 3).

## Discussion

The analysis revealed a significant increase in notifications of burnout syndrome over the years, with the highest concentration in the Southeast region, which accounted for more than half of the cases (52.81%). This scenario may be associated with the intense industrialization and urbanization of major centers such as São Paulo, which historically promote greater competitiveness and heavier workloads.^^15^^ Previous studies, such as Bakker & Costa,^^16^^ indicate that these factors heighten occupational stress levels, contributing to the classic manifestations of the syndrome: emotional exhaustion, depersonalization, and reduced professional accomplishment. Moreover, aspects such as access to health services, infrastructure, and monitoring programs may influence the number of reported cases.^^15^,^16^^

Regarding gender, the higher prevalence observed among women (71.60%) is consistent with literature, which points to greater emotional exhaustion in this group, whereas men tend to present more depersonalization. This female vulnerability may be explained by the burden of a double workday and additional social and professional pressures. However, the p-value (p = 0.27) indicates that this difference was not statistically significant, suggesting possible reporting biases or limitations in data. Thus, further studies are needed to investigate occupational and educational factors that influence the development of burnout in each gender.^^17^,^18^^

As for sociodemographic factors, the higher prevalence among White individuals and those aged 35-49 is also consistent with literature, which associates greater risk with younger workers and those with less professional experience. This may be explained by high demands and a lack of effective strategies for coping with stress at the beginning of a career. On the other hand, the lower prevalence among older adults (0.07%) may be related to a reduced workload after retirement, resulting in less exposure to occupational stressors and greater routine stability, favoring improved quality of life.^^19^,^20^^

Alcohol and other drug use was reported as infrequent in this study. Although licit and illicit substances are often used as coping mechanisms for stress, studies indicate that health workers present consumption patterns similar to the general population but are at greater risk of dependence due to increasing occupational stress. Among medical students, for example, there is evidence of higher consumption and vulnerability to dependence, driven by intense emotional strain and pressure during university training.^^21^,^22^^

Most participants reported lifetime use of psychotropic drugs, which warrants attention, especially since burnout syndrome is not classified as a mental disorder in the Diagnostic and Statistical Manual of Mental Disorders (DSM-V) but rather as an occupational phenomenon, with recommendations favoring nonpharmacological treatments. Although multidisciplinary follow-up with an emphasis on nondrug interventions is ideal, this study showed a predominance of psychotropic drug use. Such medicalization may reflect an institutional culture oriented toward quick solutions, rather than approaches centered on the individual, in addition to limitations in the availability and adherence to Integrative and Complementary Health Practices (PICS), which, although recommended by the Brazilian Ministry of Health, still face resistance and limited engagement.^^23^-^25^^

Furthermore, the risk of self-medication should be highlighted, especially among health professionals who, due to easier access, use psychotropic drugs without medical prescription. This practice has been associated with higher levels of depersonalization, with a statistically significant difference for the use of sedatives (p = 0.005). Even aware of the risks, many resort to these medications to endure the intense workload, underscoring the urgent need for prevention strategies and awareness regarding the proper use of these drugs.^^21^^

With respect to treatment, outpatient care predominated (92.11%), along with referrals to CAPS (48.96%), consistent with recommended practices that prioritize psychological follow-up, supportive therapies, and workplace interventions. The low hospitalization rate reinforces the possibility of effective outpatient management, provided that continuous follow-up is ensured. Referral to CAPS broadens multidisciplinary care, focusing on social reintegration and the prevention of complications, including the abusive use of other substances.^^24^,^26^^

The analysis of clinical outcomes indicated that most patients developed temporary disability (69.34%), which is expected in cases receiving appropriate intervention. However, the occurrence of permanent disability (0.27%) in a small group highlights the potential severity of the syndrome. This situation may be explained by difficulties in returning to work, associated with fear of reliving stressful situations, excessive perfectionism, or precarious workplace conditions, all of which compromise the physical and mental health of workers. These aspects represent important challenges that remain underexplored in literature.^^27^^

In summary, the results corroborate previous findings by demonstrating an increase in the prevalence of burnout syndrome in recent years, with greater impact in large cities and specific sociodemographic groups. However, the detailed analysis of subgroups such as race and substance use highlights the need for further research to better understand the social and cultural factors involved. Such data are essential for the development of targeted prevention and treatment strategies, including workplace interventions, manager training, and emotional support programs in health services.

## Conclusions

Burnout syndrome showed a marked increase in Brazil over the past decade, with the highest prevalence in the Southeast region, predominantly among White women aged 35-49 years. This pattern reflects the influence of factors such as regional urbanization, high workload, and the burden of women’s double workday. A significant association was observed with the use of psychotropic drug, whereas the use of alcohol, tobacco, and recreational drugs were less prevalent, indicating the need for further investigation. Most cases were managed in outpatient settings, with outcomes progressing to temporary disability. These findings underscore the impact of social and cultural determinants on the population’s mental health and reinforce the urgency of effective public policies aimed at prevention, early diagnosis, and comprehensive care, particularly in primary health care.
